# P-324. Assessing Current Knowledge and Attitudes about PrEP for HIV Prevention on Chicago’s South Side

**DOI:** 10.1093/ofid/ofaf695.543

**Published:** 2026-01-11

**Authors:** Paul Copeland, Chinwe S Udemgba, Mayra Malagon, Jordan Victorian, Ellyse Grunsten, Marie Grace Giramahoro, Jade Pagkas-Bather

**Affiliations:** University of Chicago Pritzker School of Medicine, Chicago, IL; University of Chicago Pritzker School of Medicine, Chicago, IL; University of Chicago Medicine, Chicago, Illinois; University of Chicago Medicine, Chicago, Illinois; University of Chicago Medicine, Chicago, Illinois; University of Chicago Medicine, Chicago, Illinois; The University of Chicago

## Abstract

**Background:**

Pharmacy-led pre-exposure prophylaxis (PrEP) is an emerging method to improve PrEP uptake across the United States, with few states adopting this novel strategy. This PrEP uptake modality would centralize PrEP care within pharmacies and rely on pharmacists for PrEP prescriptions, on-site laboratory draws, STI screening, and counseling services, thus eliminating the need for prescriptions and visits with medical providers. Early research among young Black sexual minority men indicated a preference for pharmacy-based or mail delivery options for antiretroviral medications, emphasizing the importance of convenience and privacy. This study examined the current knowledge and attitudes toward PrEP among patients receiving care at a federally qualified health center (FQHC) and the effectiveness of a deliberative focus group to improve PrEP knowledge.

**Figure:**
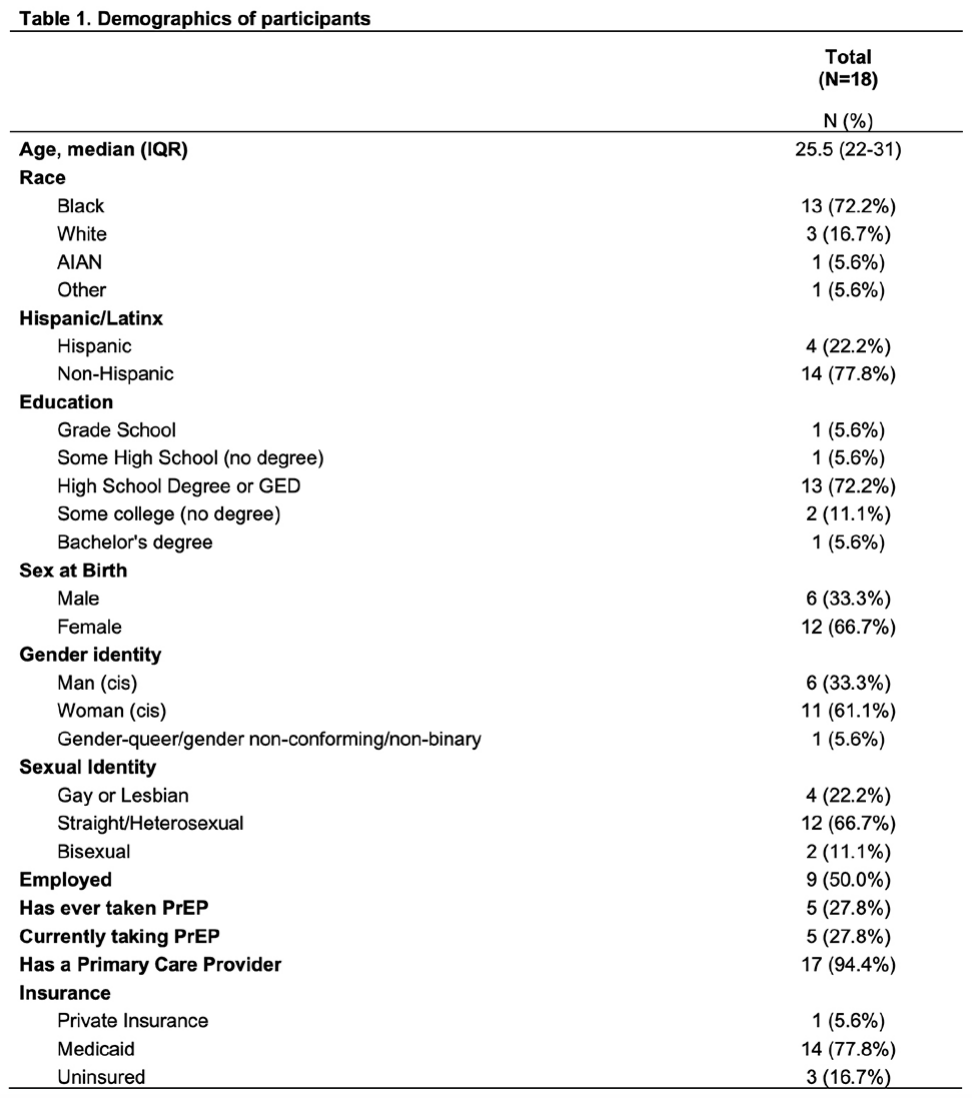

**Methods:**

Patients from a FQHC participated in a 30-minute PrEP deliberative focus group with an upfront informational session regarding PrEP eligibility, use, formulations (oral, injectable, future implants), and side effects. Surveys administered via REDCap before and after the session assessed changes in PrEP knowledge. Demographic and clinical data were taken from electronic medical records.

**Results:**

Among 18 participants, a paired t-test showed a significant increase in knowledge scores post-session (pre-test: mean=8.94; post-test: mean=10.44, p=0.006). Demographic information of participants is shown in Table 1.

**Conclusion:**

Our work demonstrates that a deliberative focus group significantly improved PrEP knowledge among patients at a FQHC in a predominantly Black community with some of the highest rates of HIV in Chicago. These findings support the feasibility of patient education initiatives within a pharmacy-led PrEP model. Our future work aims to assess acceptability, feasibility, and appropriateness of pharmacy-led PrEP from the perspectives of patients, local pharmacists, and pharmacy technicians. The results of this work will provide pre-implementation data to inform key elements for scale up of a pharmacy-led PrEP program.

**Disclosures:**

All Authors: No reported disclosures

